# Cognitive social capital for knowledge absorption in specialized environments: The path to innovation

**DOI:** 10.1016/j.heliyon.2023.e14223

**Published:** 2023-03-02

**Authors:** Eduardo Sánchez-García, Bartolomé Marco-Lajara, Javier Martínez-Falcó, Esther Poveda-Pareja

**Affiliations:** Department of Management, Faculty of Economic and Business Sciences, University of Alicante, 03690 San Vicente del Raspeig, Alicante, Spain

**Keywords:** Regional specialization, Cognitive social capital, Absorptive capacity, Innovation, PLS-SEM

## Abstract

The purpose of this research is to empirically analyze how regional specialization influence innovation in enterprises, as well as the mediation effect of absorptive capacity and cognitive social capital, as knowledge diffusion mechanisms. A questionnaire was developed and distributed, obtaining a sample of 197 energy firms in Spain. For the assessment of this data, the PLS-SEM technique, a multivariate analytical approach, has been used. The results show a positive and significant direct effect between the degree of regional specialization and the innovative performance of firms. In addition, the cognitive social capital and absorptive capacity variables show a positive and significant mediation effect in the relationship proposed, as well as between them, thus constructing a double mediation and proving to be relevant mechanisms for knowledge diffusion. Then, it is concluded that cognitive and geographical proximity matters, since enable firms to obtain resources for knowledge upgrading and innovation. Thus, it is crucial for firms to develop their capacity to absorb new external knowledge, as has been evidenced as a key factor to leverage the opportunities of the context in which firms are located. This paper has important implications for the long-standing complex debates about whether regions should develop, primarily whether they should do so through specialization or diversification.

## Highlights

• Provide empirical evidence on the drivers of firms' innovative performance.
• Emphasize the role of cognitive social capital as a key source for the development of collaborative innovations.
• Highlight the importance of developing absorptive capacity to leverage knowledge from the environment.
• Establish the benefits of regional specialization for firms in terms of knowledge and innovation.

## 1 Introduction

Today, innovation is the most powerful factor driving economic and social growth, being important to the socioeconomic development of society and touching all persons and agents that make it up [1]. The rising complexity and rapidity of the corporate environment have prompted a heightened emphasis on innovation as the key to a company's long-term success [2]. In an attempt to gain a competitive advantage, a growing number of businesses are seeking external expertise to stimulate innovation [3]. According to the categorization of innovation implementers, the vast majority of theoretical study in the field of innovation is conducted at the business level, focusing on input decision-making, output performance, and the accompanying transmission mechanisms by persons [4]. The explosion of innovation activity coinciding with times of fast economic expansion in both established and developing nations has sparked a rising interest in the environmental variables that influence innovation success [5].

While logic might predict that the rapid pace of globalization of the economy, the decreasing cost of shipping products and the development of ICT would diminish the importance of location as a critical enabler of corporate success, evidence suggests that the importance of the local environment has increased over time [6]. Therefore, the location of companies' facilities is nowadays a crucial decision that affects the features of the actors involved, which can promote the creation of location economies resulting in a relative benefit over companies located in widely scattered geographic areas. However, physical closeness does not always indicate advantages for the companies in a particular location [7]. Innovation generation and inter-organizational learning seem to require, besides geographical closeness, the presence of cognitive proximity and similar social patterns between geographically adjacent experienced economic actors belonging to a main industry, which allows for successful communication [8]. Thus, industrial agglomeration refers to those organizations located close in geographical terms, and with experience in a main sector, which usually present common cognitive and social features.

The proximity resulting from agglomeration facilitates the formation of bonds among business players belonging to a dominant sector, thus making communication easier, the generation of common objectives and values, and the effectiveness and efficiency of knowledge transfer [9]. Moreover, absorptive capacity may be understood as the foundation of creativity [10]. According to this perspective, companies that have a comparatively a stronger capacity for the absorption of new external knowledge are able to surpass whatever cognitive and technical limitations otherwise would prevent them to use external sources of information. Location in regions with a dense network of linked activity also enhances the innovative performance of businesses [11]. Moreover, it has been shown that this link is favorably mediated by the firm's pre-existing internal resources, such as social capital. To an extent, companies that have strong absorptive capacity are expected to have comparable performance in terms of innovation, despite differences in other firm parameters. Several empirical studies examine the effect of absorptive capacity in determining a firm's potential to benefit from economies of agglomeration [12]. Then, firm absorptive capacity may spur business performance in terms of innovation [13].

However, there is currently some lack of knowledge about how geographical and cognitive proximity of specialized firms relate to each other, and their role in their ability to absorb knowledge and innovate [14]. In addition, it remains unclear which of the positive externalities provided by geographical and cognitive proximity are more beneficial to the growth of enterprises and their innovative performance [15]. This research adds to the ongoing discussion over the significance of geographical and cognitive proximity of specialized firms on knowledge absorption and the development of innovations.

The main purpose is to empirically analyze the relationship between regional specialization and the innovation outcomes in enterprises, besides the mediation effect of absorptive capacity and cognitive social capital. This research adds to the literature by presenting empirical data about the significance for enterprises of locating their activities in specialized regions and its effect on cognitive social capital, absorptive capacity, and innovative performance. Then we examine the importance of the cognitive and learning capabilities of the firm to specifically take advantage of the localized knowledge spillovers expected to occur among companies located in comparable sectors.

This work complements research in adjacent fields and offers a novel research approach for future investigations. The preceding discussion raises the topic of whether geographical specialization influences innovative performance. Cognitive social capital and absorptive capacity are potential mediators of this association.

The analysis is carried out as outlined below. A questionnaire including valid and reliable scales for estimating the variables contained in the suggested model was developed and administered to the companies examined, generating 197 valid units. PLS-SEM was used to estimate the hypothesized relationship. Replies were uniquely tagged and evaluated using SmartPLS software version 3.9.

The research is structured as described below. The research hypotheses and model nomogram are derived from a literature review pertinent to the investigated variables and relationships. The methodology of the investigation is then described, followed by its findings. The study's conclusion emphasizes the need for more research into the effect of social capital and absorptive capacity on corporate innovation.

## 2 Theory and hypotheses

### 2.1 Regional specialization and innovative performance

The capacity of a company for innovation is not only limited by its borders but depends to an increasing extent on external assets available in certain regions. Currently, industrial clusters are seen as a crucial pathway to the economic success and open innovation activities [16]. Viewed from an evolutionary point of view, innovation is conceptualized as an uncertain and cumulative process [17]. As a method of risk reduction, businesses can minimize the uncertainty of innovation by engaging in an external scan in an effort to locate and acquire knowledge inputs from outside. Integration of external information is a difficult and intricate process. This is in part due to the lack of clarity of the boundaries between different capabilities and technologies, making it difficult for focused companies to effectively search for knowledge inputs from outside [18].

Clusters are associated with a number of theoretical concepts and perspectives relating to geographically integrated agglomerations of firms and other specialized entities, such as regional innovation systems and industrial districts [19]. Ranging from economic geography and spatial planning to public administration and economic development, Porter's notion of clusters has sparked debates in a variety of scholarly fields [20]. Experts and economic policymakers have shown a significant deal of interest in clusters over the last two decades, and support for clusters has become the leading regional development approach [21]. Previous research on agglomeration focused purely upon the effect of specialized firms' closeness in capturing externalities, often being reported a significant positive correlation linking firm innovation and economic performance to agglomeration economies [22].

Clusters have evolved into a contemporary type of industrial collaboration, and their inventive character is regarded as a major regional and national source of business success [23]. In the first instance, companies tend to locate their activities in locations geographically close to their competitors, perceiving that they can benefit from agglomeration effects [24]. According to empirical research, projected spillovers across comparable co-located businesses support, on average, greater economic success, and innovation in the region [25].

Clustered enterprises are characterized by a high level of specialization and complementarity [26]. Significant advantages of clustering exist in relation to innovations, technical advances, and research and development expenditures [27]. Clusters enhance the companies' productivity and innovation capacities due to the fact that these groupings of businesses have access to a comprehensive information database [28]. Furthermore, rivalry between companies in a cluster drives companies to improve and innovate in a wide range of areas related to the exercise of their activities [29].

Agglomeration economies provide firms access to a large pool of resources, especially in terms of knowledge, which can serve to boost a firm's innovation and the regional development [30]. However, closeness may sometimes stifle innovation, due to excessive competitive pressure and the potential negative externalities arising from this situation [21]. However, some research empirically demonstrates a rather good correlation among regional specialization and performance in terms of innovation [21,31]. This beneficial linkage may be limited to specific sectors, growth periods, regions and/or unique situations [32]. In light of the aforementioned, it is suggested the first hypothesis:

Hypothesis 1 (+): There is a positive and significant relationship between regional specialization and firms' innovative performance.

### 2.2 Cognitive social capital

Cognitive social capital is a concept that businesses develop through social networks. Social capital in its cognitive dimension relates to the perception and comprehension of the language, norms, codes, values, and objectives shared by the members of the social network, which, according to Zheng [33], promotes interaction between the network nodes and increases the network's effectiveness. The cognitive component demonstrates how to build and cultivate productive social interactions within a specific network or social context [34].

Some of the most influential factors identified as those that significantly contribute to the success of open innovation processes are culture, complementarity of technological resources, trust among the actors participating in the process, and belonging to a cluster [13,35]. Then, this cognitive paradigm relates to all parties' shared interpretations and value orientations in social networks. Previous research has shown that social capital facilitates firm innovation in an environment that is reasonably stable [36].

Nevertheless, the present dynamic of the environment necessitates a more in-depth examination of the elements within the control of businesses that drive their creative success. Social operation and economic transaction are lubricated by social norms and widespread trust. The greater the trust level between the members of the network, the better the tacit information exchange becomes. Pervasive trust increases networking expansion and sociality in innovation activities, creates a logical prospect of positive reciprocity in collaborative inventive activities, and reduces the development cycle of inventions [37].

A social climate of honesty and reliability favors a mutual beneficial long-term partnership for collaboration, considered a vital factor in the development of a reliable system for regional innovation [38]. On the basis of shared or comparable cognitive patterns (consensuses) and values, communication is often vigorous and fruitful. Consensus is reached by integrating the differences between the parties and engaging in cooperative sets. Through the process of achieving agreement, the cognitive pattern of the opposing stakeholder collides with the existing firm's cognition and breaks its initial cognitive boundary [39]. Afterwards, a company with a novel cognitive pattern may seek diverse information through the process of interaction, fostering corporate innovation and serving as a point of innovation diffusion [40].

Furthermore, cognitive social capital is shaped through firms shared long-term declarations, objectives, and values [41]. Therefore, this factor is crucial for firms to overcome various constraints. From this approach, the cognitive component of social capital can be defined abstractly as the degree to which a firm acknowledges the shared values and consensus of its various social network peers. Moreover, cognitive social capital may also be conceptualized in terms of interactive agreements or commonality of values among companies that make it easier to share and combine information and resources, apply novel knowledge, and create an innovation-friendly environment, thereby fostering innovation.

In terms of the connection of the mentioned variables, bonds between firms based on shared values or consensus might enhance the flow of knowledge and data [42]. Then, it is rational to assume that cognitive social capital has significantly contributed to the increase of innovative performance of organizations. Accordingly, this study proposes the following hypothesis:

Hypothesis 2 (+): Firms' cognitive social capital exerts a mediating effect on the relationship between regional specialization and firms' innovative performance.

### 2.3 Absorptive capacity

In the field of strategic management, the concept of absorptive capacity has been recognized as an essential factor that allows firms to recognize novel knowledge from the outside, fully assimilate it and apply it for commercial purposes [43] and, therefore, for the enhancement of their innovation activities; thus, it has been regarded as an important factor to foster innovative performance [44]. Diverse conceptualizations of absorptive capacity have arisen after departure from the original definition [45].

Firms' knowledge absorptive skills have received much attention especially since the discovery carried out by March and Simon [46], which found that most creative firms prefer to monitor and learn from other firms rather than to produce new knowledge in isolation, using just the company's own resources. Later, Cohen and Levinthal's [47] key work on learning and innovation refined this notion further and argued that businesses often have an absorptive capacity that reflects their ability to recognize, integrate, and use externally sourced information. Other writers, such as Lane et al. [48], describe it as a company's capacity to employ external information via exploratory, transformational, and exploitative learning processes, applied sequentially. This work adopts the notion where absorptive capacity is understood as the capability to recognize, integrate, transform, and utilize external knowledge [49].

Scaringella and Burtschell [50] determined that this skill may be employed at the individual, organizational and regional tiers, with collective learning and information sharing helping companies to achieve better results. A company's capacity to acquire innovative information is contingent on its search techniques and knowledge functions [51]. Therefore, they must commit both resources and skills to maintain a high degree of absorbency. Previous research has shown that the adoption of innovative techniques relies on the company's ability to gather, disseminate and leverage internal and external information and knowledge [52]. The absorptive power of enterprises to detect, acquire, comprehend, and utilize external information directly may influence their capacity to develop innovations, being both capacities developed through a dynamic learning processes [19,53].

Under the knowledge-based perspective, a company's present level of knowledge has a crucial bearing on its further knowledge development [54]. In the literature on agglomeration, however, the extent to which firms benefit from localization economies depends, to a large extent, on the capacity of firms in the region to absorb new knowledge. Then, the connection between the firm's current stock of knowledge and its ability to seek and incorporate new knowledge inputs from the outside into their own production cycles is underlined [55]. The absorptive capacity of enterprises is no longer tied to past relevant knowledge alone but may also be impacted by the degree of regional specialization where firms establish their operations. As a multidimensional concept, incorporates learning processes at the organizational, individual, and social levels [56]. Valuing, absorbing, and exploiting external information requires both individual and collective learning through social processes, as those mentioned above [57].

Thus, absorptive capacity enables organizations to efficiently and effectively absorb new information from the environment and grow their knowledge base in a cyclical process, which might boost their innovative performance. Particularly in specialized locations, organizations have found absorptive capacity valuable for using the particular knowledge of their environment to increase innovative performance [58]. These factors indicate the following hypothesis:

Hypothesis 3 (+): Firms' absorptive capacity exerts a mediating effect on the relationship between regional specialization and innovative performance.

### 2.4 Examining the relationship between regional specialization, cognitive social capital, absorptive capacity, and innovative performance

The methodical practice of innovation emerges from the examination of the environment in search of possibilities and necessitates a continual influx of fresh information [19,59]. According to Parra-Requena et al. [60], how smoothly and effectively the knowledge is shared between the different entities within the context of a cluster depends on the robustness of networks, and the existence of mutual trust and common values, besides the capacity of firms to acquire and exploit it. Although research on these aspects dates back many decades, their combined analysis is mostly a product of the past two decades, during which academics' interest in this area of study has grown tremendously [61]. Belonging to a specialized area encourages the process and knowledge expertise of the businesses involved in the principal sector, besides those engaged in auxiliary industries [62].

The positioning of companies in specialized locations is advantageous in terms of organizational procedures, expertise, and access to strategic resources [63]. The intricate network of relationships resulting from the distinctive pattern of distribution of these locations, fostered at the same time by closeness, promotes the sharing of knowledge and information, mostly tacit and primarily conveyed by socialization among local agents, which allows businesses learning from each other and supporting a continuous improvement process. In every informal process of learning, socialization is vital to the sharing and merging of knowledge [64].

In this way, trust, culture, and a sense of belonging, among other things, may influence the level of incentive of a network's members to engage in the cooperative dynamics of the network and, subsequently, the access to the potential resources it contains [19,63]. Consequently, the geographical closeness of businesses associated with a primary sector may facilitate the growth of their cognitive social capital, particularly as these participants tend to have similar values, aims, beliefs, culture, and corporate vision. According to Parra-Requena et al. [60], in the setting of a cluster, the flow of external information may boost the innovative performance of businesses, thus firms that are able to get a bigger quantity of relevant external knowledge would increase their innovative performance.

Positive cognitive social capital firms regularly engage in consensus-sharing with stakeholders, allowing them to successfully use, soak, and export consensus-based information, due to the fact that is widely seen as secure, pleasant, and seamless [39]. In contrast, robust absorptive capacity reduces the cost of acquire, convert, and leverage new knowledge, hence contributing to the growth of firms' knowledge stocks. Through the absorption of information, businesses may produce new strategies for optimizing current processes, such as product creation, manufacturing, and marketing. Therefore, absorptive capacity is a crucial part in the knowledge generation processes of firms [62].

A wide knowledge base is the foundation for the development of absorptive capacity [65]. In addition, knowledge searchers may comprehend, integrate, and change new information by interacting with sources of knowledge [66]. Establishing an efficient way of seeking knowledge thereby helps the knowledge base to grow. Consistent with previous research, it is anticipated that firms situated in regions with greater degrees of regional specialization would demonstrate superior innovative performance, and that cognitive social capital and absorptive capacity will positively mediate this positive association.

**Fig. 1:**
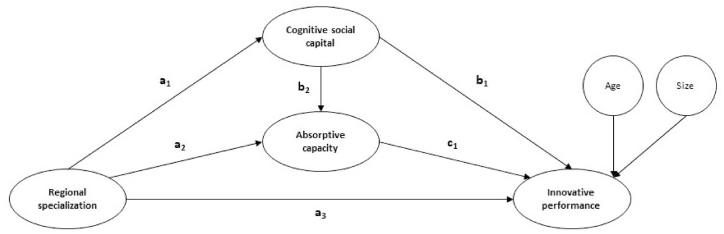
Nomogram of the proposed model.

Based on the preceding, it can be concluded that cognitive and organizational characteristics, in addition to those connected to the degree of specialization of the businesses' local environment, are among the most important determinants of absorptive capacity. Localization can improve the innovative performance of companies as a result of the impact of proximity and of being part of a particular social and economic context, where players have similar cognitive features, particularly how they conduct and interact with each other, and how they understand business. Then, is proposed the fourth hypothesis, and the nomogram of the model is showed in Fig. 1.

Hypothesis 4 (+): There is a double mediation of the firms' cognitive social capital and firms' absorptive capacity in the relationship between regional specialization and firms' innovative performance.

H1 = a3: Regional specialization → Innovative performance.
H2 = a1 × b1: Regional specialization → Cognitive social capital → Innovative performance.
H3 = a2 × c1: Regional specialization → Absorptive capacity → Innovative performance.
H4 = a1 × b2 × c1: Regional specialization → Cognitive social capital → Absorptive capacity → Innovative performance.

## 3 Methodology

### 3.1 Population and sample

The population under examination consists of Spanish enterprises operating in the domain of power supply. According to the SABI database, in 2019 there were 13,339 firms functioning in Spain. The sample includes 197 operational Spanish businesses. Despite employing just 2% of the overall workforce in Spain, this sector contributed 13.8% of the gross added value and 9.4% of industrial output in 2019, making it the second most significant sector. Moreover, was the sector with the highest employee productivity (466,500 euros on average).

### 3.2 Data collection and measurement of variables

To obtain primary data, a questionnaire was designed and distributed to the companies of the population under study. The tool used for the design and distribution of the questionnaire was the “Qualtrics” software. In addition to SABI, the “Empresite” database, which is available on the Internet, was used to obtain the contact data of all the companies in the sector. The questionnaire was addressed to the CEO of the companies, who was considered to have a broad knowledge of the general functioning of the organization, as well as of its main decision-making bodies. The questionnaires were distributed by e-mail, using Microsoft Outlook software.

The questionnaire distribution technique spanned four months, from September to December 2020, and included many reminders and phone calls in addition to the initial mailing to encourage participants in the study. After evaluating the statistical validity of the completed surveys and deleting those deemed invalid (due to a substantial amount of lost data, patterns of response, or single-value responses), 197 valid replies were found. The minimum sample size required was assessed using the minimum R^2^ method established by Hair et al. [67] and the software Gpower, in its version 3.1, obtaining largely favorable results. Non-response bias has been assessed by comparing the responses of the first and last waves, as established by Armstrong and Overton [68].

Regional specialization (independent variable): It is a formative variable and is measured through the level of industrial concentration in every Spanish province. Agglomeration is quantified in many ways in the agglomeration literature. Some analyses utilize the density of enterprises in each sector and geographic region [[69], [70], [71]], whilst others use employment data [72,73]. They have been utilized to compute this variable. This form was extensively utilized in several studies on clusters. Although they are basic indicators, they make it easy to establish the regional distribution of firms and workers in a certain sector in a straightforward and understandable manner. As territorial units of study, we used Spain's fifty provinces and two autonomous cities. Using the following coefficient, the degree of regional specialization in the sector was calculated, relative to the national average [[69], [70], [71], [72], [73]].

RS = (a:b):(c:d)

being RS: degree of regional specialization. a: units of the sector in each province. b: total units in each province. c: units of the sector existing in the whole country. d: total units existing in the whole country.

This factor must be understood in the following manner: Regions with a value higher than one have a larger proportion of workers or businesses belonging to the sector analyzed than the average calculated for the whole country. The higher the value of a province, the more concentrated it is the sector in the territory.

The innovative performance (dependent variable) was evaluated using a 7-point Likert and thirteen-item scale. On the basis of the study of Prajogo and Ahmed [74] and Škerlavaj et al. [75], were used validated scales comprised of five (product), four (process), three (marketing), and one (management) items.

Cognitive social capital (mediating variable). Based on the dimensions of Nahapiet and Ghoshal, the measurement scale is a 7-point Likert scale. It was built based on the study of Parra-Requena et al. [60] and has seven components.

Absorptive capacity (mediating variable): 14 items have been developed and dispersed in each step (acquisition, assimilation, transformation, and exploitation) for the assessment of this variable by Zahra and George [49], based on the work by Flatten et al. [76]. As with the other factors, a 7-point Likert scale was used. According to Finstad [77], it has been shown that 7-point Likert items are more precise, user-friendly, and reflective of a respondent's real opinion. Even when compared to higher-order questions, 7-point items seem to be the optimal answer for questionnaires used in usability studies.

### 3.3 Analysis technique

To evaluate the hypotheses, we used the multivariate second-generation partial least squares, the PLS-SEM technique, multivariate analytical approach. A great number of researchers in the field of strategic business management have put their focus on this technique [78]. In this instance, version 3.9 of SmartPLS was employed [79]. According to Hair et al. [80], this method is appropriate for predictive analytics, particularly in the social sciences, due to the latent character of the variables considered in this field. Moreover, it facilitates the evaluation of models with latent linear connections between variables. The PLS-SEM approach aims to maximize variance [81]. According to these authors, this approach is especially appropriate for social science research due to the aforementioned requirements.

Martinez-Ávila and Fierro-Moreno [82] demonstrated that the PLS-SEM method is more versatile and robust than conventional techniques. This method has been selected for several reasons. The predictive nature of the investigation supports its utilization [83]. Besides, it helps the estimation of complicated models, low sample sizes in small populations and data that are not regularly distributed [80]. Lastly, the PLS approach allows to efficiently estimate second order variables, such as those incorporated in the model proposed [84].

## 4 Analysis of data and results

### 4.1 First phase of analysis

To determine the specializations’ degree of each of the Spanish provinces. In this regard, results are showed in Table 1, in which can be appreciated the extent to which each region is specialized in the sector under study, measured by the concentration of firms and employees. Also displayed is the distribution based on the degree of agglomeration of the sector in the province in which it is located, compared to the country average.

**Table 1:** Distribution of the sample in relative and absolute terms.

Coefficient	Regional specialization	Firms	% of the Sample
Employees	Higher than the national average	114 companies	57.87%
	Lower than the national average	83 companies	42.13%
Companies	Higher than the national average	112 companies	56.85%
	Lower than the national average	85 companies	43.15%

Source: Own elaboration.

In addition, in Table 2 it is exposed the distribution of the population in comparison with the sample, depending on whether it is located in a specialized area or not. As can be seen, the population and sample data reveal a clear similarity between these two groups.

**Table 2:** Population and sample distribution.

Coefficient	Regional specialization	% population	% sample
Employees	Higher than the national average	66.09%	57.87%
	Lower than the national average	33.91%	42.13%
Companies	Higher than the national average	66.62%	56.85%
	Lower than the national average	33.38%	43.15%

Source: Own elaboration.

**Fig. 2:**
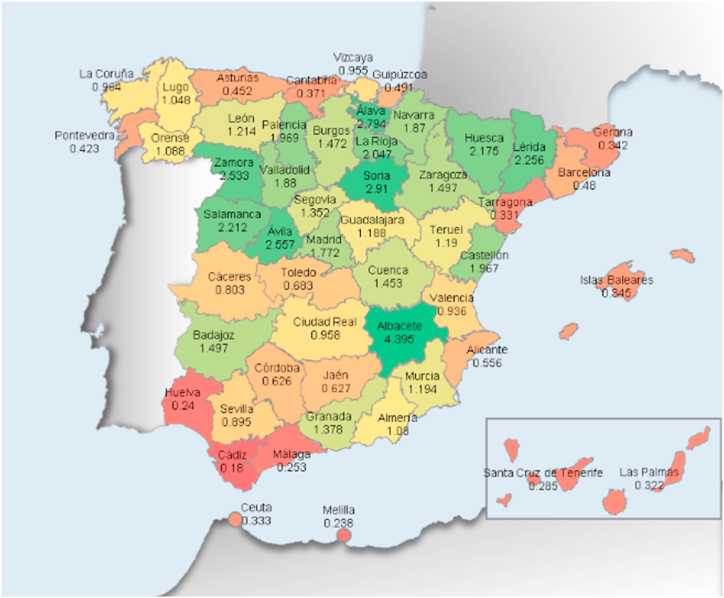
In Spain, the degree of regional specialization by provinces, measured by the presence of energy firms.

In more detail, Fig. 2 illustrates the regional specialization ratio in terms of the volume of companies in the sector.

**Fig. 3:**
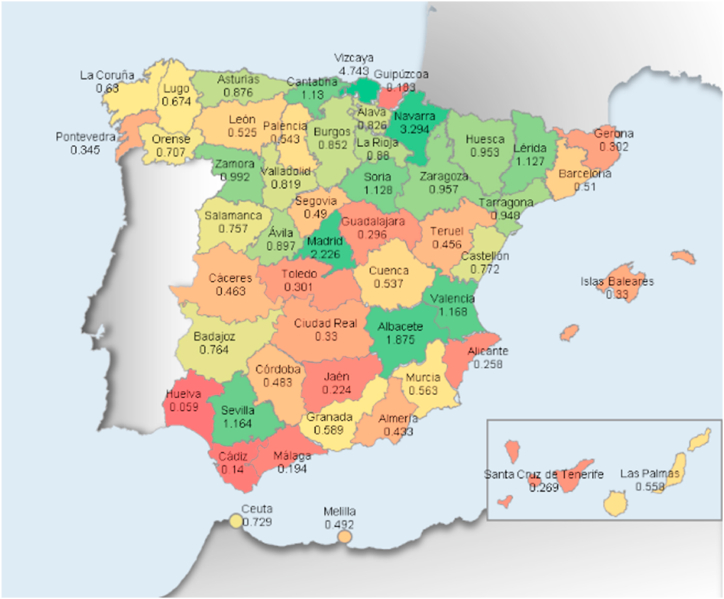
In Spain, the degree of regional specialization by provinces, measured by the presence of employment in the energy sector.

Related to this, Fig. 3 shows the same procedure, but regarding to employees’ concentration. A color spectrum has been devised, ranging from dark red for regional specialization levels significantly below to green for those above the average of the whole country. The remaining colors represent intermediate stages. In Table 3, a basic statistical description of the questionnaire results is provided.

**Table 3:** Descriptive statistic of the data obtained in the questionnaire.

	Mean	Min	Max	S.D.
Regional specialization	1.086	0.059	4.743	0.928
Cognitive social capital	4.868	1	7	1.399
Absorptive capacity	5.067	1	7	1.708
Innovative performance	4.809	1	7	1.590

Source: Own elaboration. Note: S.D.: Standard deviation.

### 4.2 Second phase of analysis

In the second phase of analysis, it is proposed a model that comprises multiple components. These are composed of several interconnected elements that may be evaluated as a single theoretical concept [85]. It is necessary to carry out a preliminary test to calculate the first-order latent variables, to use when developing the model with the second-order constructs in a subsequent research [86]. PLS is an outstanding process tool [87]. In social science investigations, this let to produce second-order variables endogenously inside the structural model [88]. Standardized Root Mean Square Residual [SRMSR] values of 0.061 > 0.08 for both the saturated and estimated final models indicate an acceptable match [89].

Distinct criteria must be applied based on whether the relevant concept is formative or reflective to assess measurement models [80,90]. Regional specialization is a formative variable. Then, the following process must be followed to evaluate it. In this first step, which is also called “redundancy analysis” and determines the convergent validity of the model, the degree of correlation between the different measures of the same construct is assessed by using different indicators [91,92]. For this, the formative latent variable is used as an exogenous variable, which acts as a predictor of another endogenous con-struct that uses other indicators of a reflective nature.

Even though, in general, the utilization of individual indicators when using the PLS technique is not recommendable, in the case of redundancy assessment it is appropriate because with the aim of having a comparative standard, the objective of redundancy analysis is to capture the fundamental elements of the construct, rather than its total content [80]. Thus, this item, called “Sec.P.GDP”, represents the percentage contribution of the studied sector to the province GDP, using secondary data from the SABI and the National Institute of Statistics databases.^1^

They both, reflective and formative indicators' path coefficient reach a value of 0.916 > 0.8, and the R^2^ 0.839 > 0.5. This, according to Hair et al. [80], implies that the model passes the convergent validity condition. The VIF result of 1.192 < 3 indicates that the formative indicators’ collinearity is at optimal levels [93]. Subsequently, it is evaluated the relevance and significance of the reflective indicators. Having run the full-mode bootstrapping process on 5000 random subsamples, were assessed the external loadings of the formative indicators, finding values that indicate a great contribution to the construct.

To assess the reflective measurement model, it must be investigated its internal consistency, convergent validity, and discriminant validity [80]. Cronbach's alpha (α), composite reliability (ρc), and Dijkstra-rho Henseler's (ρA) are utilized, according to these authors. As seen in Table 4, every outcome is significantly greater than 0.7 [80,91,94]. The size of the external loadings (λ), and the Average Variance Extracted (AVE), are used to assess the reliability of indicators, which refers to the total mean value of the squared loadings of the indicators belonging to the same construct [80]. In addition, convergent validity is assessed through the analysis of the external loadings, checking that they have a value more than 0.707 and that the AVE is higher than 0.5 [80,95].

**Table 4:** Assessment of internal consistency and convergent validity.

Internal consistency and convergent validity				
	Cronbach's Alpha	rho_A	Composite reliability	Average extracted variance (AVE)
Absorptive capacity	0.819	0.840	0.880	0.647
Cognitive social capital	0.871	0.872	0.901	0.565
Innovative performance	0.849	0.850	0.898	0.689
External loads	A.C.	I.P.	C.S.C.	VIF values
AC acquisition	0.737			1.556
AC assimilation	0.824			1.786
AC transformation	0.786			1.961
AC exploitation	0.866			1.643
Cognitive social capital_1			0.722	1.605
Cognitive social capital_2			0.740	1.762
Cognitive social capital_3			0.758	1.888
Cognitive social capital_4			0.756	1.820
Cognitive social capital_5			0.725	1.796
Cognitive social capital_6			0.791	2.272
Cognitive social capital_7			0.766	2.015
IP management		0.816		1.829
IP marketing		0.791		1.673
IP process		0.856		2.238
IP product		0.855		2.224

Source: Own elaboration.

Note: C.S.C.: Cognitive social capital; A.C.: Absorptive capacity; I.P.: Innovative performance.

The Heterotrait-Monotrait Ratio (HTMT) is a more effective tool than the Fornell and Larcker and cross-loading analysis for determining discriminant validity difficulties, despite the fact that both prerequisites are met [95]. Kline [96] states that the HTMT ratio must be less than 0.85. The model largely satisfies this criterion, as demonstrated by Table 5.

**Table 5:** Evaluation of discriminant validity.

Discriminant validity			
FORNELL-LARCKER	A.C.	C.S.C.	I.P.
Absorptive capacity	0.805		
Cognitive social capital	0.636	0.751	
Innovative performance	0.642	0.590	0.830
HTMT	A.C.	C.S.C.	I.P.
Absorptive capacity			
Cognitive social capital	0.736		
Innovative performance	0.758	0.681	

Source: Own elaboration.

Note: C.S.C.: Cognitive social capital; A.C.: Absorptive capacity; I.P.: Innovative performance.

## 5 Results

The assessment of the structural model allows to assess the model's predictive power and the nature of the model's numerous latent variables' interrelationships, and so to evaluate the hypotheses provided within the theoretical framework. The evaluation of the structural model is undertaken according to the method outlined by Hair et al. [80]. In the first step, an Algorithm PLS analysis is performed to assess the degree of collinearity between the predicted constructs, with the VIF value kept below 3 [93].

The path coefficients of the established associations are then calculated by executing the bootstrapping procedure in full mode with 5000 random subsamples and a 99% confidence interval. These coefficients, whose values range from 0 to 1, reflect the extent to which a change in the value of the source variable affects the value of the target variable. The R^2^ coefficients are then used to evaluate the predictive power of the model for each variable. According to Hair et al. [80], R^2^ values of 0.25, 0.50, and 0.75 are weak, moderate, and significant, respectively. Next, the ƒ^2^ size of the effects is analyzed to assess the influence of each exogenous construct on the R^2^ value of the related endogenous latent variable. If the ƒ^2^ value is close to 0.02, 0.15, or 0.35, it is classified as small, moderate, or large effect [80]. Lastly, the blindfolding method is utilized to examine the cross-validation redundancy index Q^2^, which reflects the predictive significance of the model with respect to each endogenous component. Q^2^ values greater than zero, 0.25, and 0.50, respectively, indicate low, moderate, and substantial predictive significance [90].

**Fig. 4:**
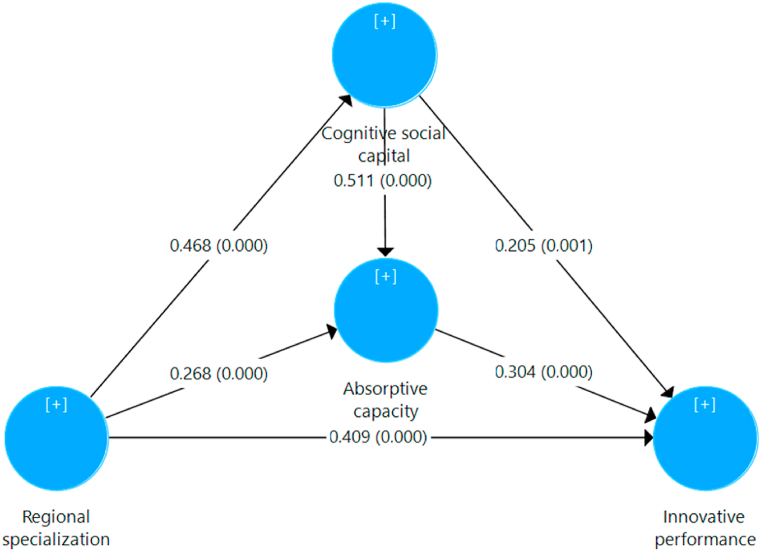
Nomogram of the model with path coefficients.

In the subsequent analysis, the omission distance D was determined by the constraint that the sample size cannot be divided by this number to yield an integer. Consequently, the D value selected was 7 [Sample size = 197]. According to Hair et al. [80], the significance and importance of the connections, collinearity, the coefficients of determination’ values (R^2^), effect size (ƒ^2^), and predictive significance (Q^2^) must be evaluated. The direct effects of carrying out the bootstrapping technique are shown in Fig. 4. Furthermore, the direct and indirect effects are shown in Table 6, Table 7, respectively.

**Table 6:** Summary of direct effects.

Structural path	Coef. (β)	S.D.	P-values	99% C.I.	Results
C.S.C. → I.P.	0.205**	0.064	0.001	[0.069–0.318]**	
R.S. → C.S.C.	0.468**	0.050	0.000	[0.374–0.567]**	
R.S. → I.P.	0.409**	0.055	0.000	[0.266–0.482]**	H1 supported
R.S. → A.C.	0.268**	0.048	0.000	[0.176–0.364]**	
C.S.C. → A.C.	0.511**	0.055	0.000	[0.403–0.618]**	
A.C. → I.P.	0.304**	0.064	0.000	[0.185–0.434]**	

Source: Own elaboration.

Note: Coef.: Coefficient; S.D.: Standard deviation; C.I.: Confidence interval; R.S.: Regional Specialization; I.P.: Innovative performance; C.S.C.: Cognitive social capital; A.C.: Absorptive capacity; ** Statistically significant at 1%.

**Table 7:** Summary of indirect effects.

Total effect of R.S on I.P.		Direct effect of R.S. on I.P.		Indirect effect of R.S. on I.P.			Conclusion
Coef. (β)	T value	Coef. (β)	T value	Point estimated		C.I. 99%.	
0.659**	19.634	0.409**	7.374	Total	0.250		
				H2 = a_1_ × b_1_	0.096**	[0.037–0.169]	H2 supported
				H3 = a_2_ × c_1_	0.081**	[0.042–0.135]	H3 supported
				H4 = a_1_ × b_2_ × c_1_	0.073**	[0.039–0.114]	H4 supported

Source: Own elaboration.

Note: Coef.: Coefficient; C.I.: Confidence interval; R.S.: Regional Specialization; I.P.: Innovative performance; ** Statistically significant at 1%.

The data analysis indicates that there is no collinearity, as all VIF values are less than 3 [93]. Regional specialization has a positive and statistically significant effect on the innovative performance of firms [0.409, p = 0.000]. In addition, the variables “Cognitive social capital” and “Absorptive capacity” mediate a positive and statistically significant indirect effect in this relationship [0.096, p = 0.000] and [0.081, p = 0.000] respectively. Furthermore, they both exerts a double mediation effect [0.073, p = 0.000]. The proposed model explains 21.9%, 46%, and 61.7% of the variance of the “Cognitive social capital,” “Absorptive capacity” and “Innovative Performance” components, respectively.

The “Regional specialization” contribution to the R^2^ value of the endogenous latent variables “Cognitive social capital,” “Absorptive capacity” and “Innovative Performance” (ƒ^2^) is moderate [0.280, 0.104, 0.259] respectively [97]. Finally, the Q^2^ values of the endogenous variables’ “Cognitive social capital”, “Absorptive capacity” and “Innovative Performance” are 0.118, 0.284 and 0.396, respectively, which indicates that the model has a moderate predictive relevance on the mentioned variables [90].

Then the four hypotheses proposed are accepted.

Hypothesis 1 (+): There is a positive and significant relationship between regional specialization and firms' innovative performance.
Hypothesis 2 (+): Firms' cognitive social capital exerts a mediating effect on the relationship between regional specialization and firms' innovative performance.
Hypothesis 3 (+): Firms' absorptive capacity exerts a mediating effect on the relationship between regional specialization and innovative performance.
Hypothesis 4 (+): There is a double mediation of the firms' cognitive social capital and firms' absorptive capacity in the relationship between regional specialization and firms' innovative performance.

## 6 Discussion

This work adds to the body of research on firms' location and innovation by focusing on the cognitive proximity of enterprises and their internal capacity to absorb new knowledge in specialized contexts. By analyzing geographical and cognitive closeness, we enhance our understanding of their connection and influence on the assimilation of new knowledge and the creation of innovations. In this view, membership in a specialized region is insufficient to properly harness the innovative potential of enterprises, being cognitive closeness and the firms’ absorptive capacity crucial components for maximizing the benefits of these specialized contexts. Then, the results of this research are significant and contribute to the literature on clusters, knowledge, social capital, and innovation. This study contributes to the research on energy firms' innovative performance by examining the positive and significant relationship between regional specialization and firms' innovative performance. Over the last few years several studies have linked location of companies in specialized environments with a positive and negative effect on their performance [98], while others indicate the existence of a curvilinear relationship or even the absence of it [99]. However, there may be other variables that may influence this relationship, especially in view of the rapidly changing environment in which companies must operate. Then, there is a need to analyze other contingent factors that may influence this relationship.

The findings of this study indicate that, nowadays, firms located in specialized environments can generate higher innovative performance. In this case, the mediating role of cognitive proximity and absorptive capacity of firms was analyzed, being empirically evidenced their contribution to the relationship between the location of firms in specialized contexts and the effective improvement of their innovative performance. From a knowledge and cognitive perspective, this research deepens the analysis of the links between proximity and innovation performance, considering the theoretical difference between cognitive and geographical closeness. This research considers the degree of cognitive closeness at the organizational level which might help to explain the unequal access to important information and knowledge across enterprises in clusters. In addition, the concept of absorptive capacity was linked in the literature to the so-called dynamic capabilities, being one of the most important to be developed by companies. In this vein, while internal sources of knowledge are crucial, external sources are also required for a company to achieve the appropriate degree of innovativeness and sustain an exceptional capacity for introducing innovations, which justify the need for deepen the analysis on the role of absorptive capacity of firms in this context.

This paper has also important implications for management. The geographical and cognitive proximity between firms and other surrounding knowledge sources may help them to access and exploit new knowledge that, along with internally accessible information and ideas, could enhance the innovation performance of the firms. As a result, managers must consider the influence of location on their individual performance, as well as the cognitive proximity of the company to its stakeholders, especially those located geographically close to the company, when designing and implementing their strategies. Consequently, managers should pay special attention to establishing a common language and set of values to strengthen their relationships when cultural differences exist between business units. Moreover, in this line, they must be aware of the importance of promoting the development of their capacity to absorb new knowledge, not only by hiring suitable personnel, but also by creating an internal environment favorable to the generation of ideas, interaction, and cooperation, so as to favor the dissemination and exploitation of the knowledge within the company. In this way, by accessing new sources of knowledge, the company will be able to assimilate it, incorporate it into its current knowledge base and exploit it effectively to achieve the established objectives, especially in terms of innovation. Regarding to this, it is essential to consider the influence of these variables along with the absorptive capacity of organizations on the capacity of firms to develop innovations. In this work has been empirically evidenced that absorptive capacity enhances the exploitation of geographical and cognitive closeness of specialized agents with innovative purposes. By improving the understanding of how and why location influences knowledge transfers and innovation, strategic decision making in the management of a firm's location, knowledge, and networks can be facilitated. In addition to context features, the significance of cognitive social capital and absorptive capacity is emphasized, demonstrating how crucial it is to leverage the internal characteristics of the firms to improve their innovative performance.

## 7 Conclusions

This research adds to the clarification and expansion of knowledge about the principles driving regional specialization, cognitive social capital, and absorptive capacity to boost innovative performance. The results show a positive and statistically significant direct relationship between regional specialization and the innovation performance of firms, as well as an indirect link through the mediating variables cognitive social capital and absorptive capacity (hypotheses 1, 2, and 3, respectively). Thus, cognitive and geographical proximity matters, but the capacity of firms to absorb new knowledge may be a key factor to maximize their exploitation to foster the innovation performance of the firms. In this sense, it has been empirically evidenced that both mediating variables seem to be significant factors in enhancing the innovation performance of organizations. Then, cognitive proximity and a strong capacity to absorb new knowledge are required to successfully harness all the valuable resources within the firm's reach for this purpose, as a double mediation of these variables in the established relationship has been revealed (Hypothesis 4).

These findings suggest that the research is relevant to both academics and managers based on the most recent data. Locating the activity in specialized regions enables firms to obtain resources for knowledge upgrading and innovation, and the competitive pressure motivates them to increase their efforts continually in this way to enhance their competitiveness. In such regions, product, process, marketing, and managerial innovations are developed through collaborative actions, and firms are embedded in a larger cultural and institutional framework, in which geographical and cognitive proximity establish an appropriate framework for action to the diffusion of knowledge and the establishment of trusting relationships in favor of the achievement of common objectives which considerably contribute to improve the innovation performance of firms. Then, proximity is more than simply an issue of physical distance.

Knowledge sharing usually needs a high level of mutual trust and comprehension, which is not only tied to language, but also to shared values, culture, vision, and objectives. The advantages of closeness may be converted into a spatial agglomeration force for businesses involved in interaction activities, some of which may include learning. This cognitive proximity can favor the development of efficient and effective actions of collaboration, especially in terms of knowledge. In regions where a certain type of economic activity takes hold, knowledge tends to specialize, become rooted and spread throughout the territory. It may be very difficult, if not impossible, to properly transfer all intra- and inter-firm economic features from one setting to another. Thus, agglomerations of linked economic activity are not only relics of once cost-effective spatial arrangements but are presently being reproduced due to an increased desire for quick knowledge transfer between businesses. Thus, it is this desire for valuable new knowledge, coupled with geographic and cognitive proximity, that seems to foster the absorptive capacity of firms in specialized regions.

In sum, this study demonstrates the significance of networks of useful agents and the ability to assimilate new information as innovation drivers for firms. They may have access to potential sources of vital resources and talents, including new information. Then, this paper has important implications for the long-standing complex debates about whether regions should develop, primarily whether they should do so through specialization or diversification.

## 8 Limitations and further research

Regarding to the limitations, this investigation has been carried out in the energy sector. Then, its application can be extended to other industrial sectors. Furthermore, there may be other relevant variables that influence the relationship between regional specialization and the innovative performance of firms, as the entrepreneurial orientation of companies, their level of dynamic capabilities beyond their capacity to absorb new knowledge, or other dimensions of proximity, as organizational, social, or institutional. Then, although this research provides empirical evidence on the importance of geographic and cognitive proximity of specialized agents and their capacity to absorb new knowledge to drive their innovative performance, there are other related variables that may influence this relationship. Therefore, these limitations could serve as a basis for future research.

## Author contribution statement

Eduardo Sánchez-García, Ph.D.: Conceived and designed the experiments; Performed the experiments; Analyzed and interpreted the data; Contributed reagents, materials, analysis tools or data; Wrote the paper.

Bartolome Marco-Lajara, Ph.D.: Conceived and designed the experiments; Performed the experiments; Contributed reagents, materials, analysis tools or data; Wrote the paper.

Javier Martínez-Falcó, Ph.D.: Performed the experiments; Contributed reagents, materials, analysis tools or data; Wrote the paper.

Esther Poveda-Pareja, Ph.D.: Analyzed and interpreted the data; Wrote the paper.

## Funding statement

This research did not receive any specific grant from funding agencies in the public, commercial, or not-for-profit sectors.

## Data availability statement

Data will be made available on request.

## Declaration of interest's statement

The authors declare no competing interests.

## Figures and Tables

**Table:** 

Concept	Items	F.O.V.	Definition	Measurement
**Regional specialization**	RS1		Degree of regional agglomeration of employment in the sector	Coefficient = 1: national average//Coefficient > or <1: higher of lower than national average, respectively
	RS2		Degree of regional agglomeration of firms in the sector	
**Cognitive social capital**	CSC1		We share the same ambition and vision as our contacts.	Likert scale (−3 = Strongly disagree; +3 = Strongly agree).
	CSC2		My company shows enthusiasm in the pursuit of objectives and missions common to the members of its relational network, with whom it shares its goals and objectives.	
	CSC3		We understand our contacts' strategy and needs.	
	CSC4		My firm's employees and my contacts' employees have positive attitudes towards a cooperative relationship.	
	CSC5		My firm and my contacts tend to agree on how to make the relationship work.	
	CSC6		The business practices and operational mechanisms of your contacts are similar to yours.	
	CSC7		The corporate culture and management style of your contacts is similar to yours.	
**Absorptive capacity**	AC1	Acqu.	The search for relevant information concerning our industry is every-day business in our company.	Likert scale (−3 = Strongly disagree; +3 = Strongly agree).
	AC2		Our management motivates the employees to use information sources within our industry.	
	AC3		Our management expects that the employees deal with information beyond our industry.	
	AC4	Assi.	In our company ideas and concepts are communicated cross-departmental.	
	AC5		Our management emphasizes cross-departmental support to solve problems.	
	AC6		In our company there is a quick information flow, e.g., if a business unit obtains important information.	
	AC7		Our management demands periodical cross-departmental meetings to interchange new developments, problems, and achievements.	
	AC8	Tran.	Our employees have the ability to structure and to use collected knowledge.	
	AC9		Our employees are used to absorb new knowledge as well as to prepare it for further purposes and to make it available.	
	AC10		Our employees successfully link existing knowledge with new insights.	
	AC11		Our employees are able to apply new knowledge in their practical work.	
	AC12	Expl.	Our management supports the development of prototypes.	
	AC13		Our company regularly reconsiders technologies and adapts them accordant to new knowledge.	
	AC14		Our company has the ability to work in a more effective way by adopting new technologies.	
**Innovative performance**	IP1	Product	Degree of novelty of our new products.	Likert scale (−3 = Far inferior relative to my competitors; +3 = Far superior relative to my competitors).
	IP2		Use of the latest technological innovations in the new products developed by my company.	
	IP3		Speed of new product development.	
	IP4		Number of new products introduced by my company in the market.	
	IP5		Number of our new products that are new to the market (they are the first to be launched on the market).	
	IP6	Proccess	Level of technological competitiveness of my company.	
	IP7		Speed with which the latest technological innovations are adopted in our processes.	
	IP8		Degree to which the technology used in our processes is up to date or new.	
	IP9		Pace of updating our processes, techniques, and technologies.	
	IP10	Mark.	In my company, the development of new distribution channels for products and services is an ongoing process.	Likert scale (−3 = Strongly disagree; +3 = Strongly agree).
	IP11		In my company, customer suggestions or complaints are handled with urgency and attention.	
	IP12		My company develops better marketing innovations than its competitors.	
	IP13	Manag.	My company constantly emphasizes and introduces management innovations.	

Note: Acqu: Acquisition; Assi: Assimilation; Tran: Transformation; Expl: Exploitation; Mark: Marketing; Manag: Management.

## Footnotes

1 Sistema de Análisis de Balances Ibéricos and National Statistics Institute.

## References

[1] OECD (2018).

[2] Moretti F., Biancardi D. (2020). Inbound open innovation and firm performance. J. Innov. Knowl..

[3] Chesbrough H. (2020). To recover faster from Covid-19, open up: managerial implications from an open innovation perspective. Ind. Market. Manag..

[4] Acharya V., Xu Z. (2017). Financial dependence and innovation: the case of public versus private firms. J. Financ. Econ..

[5] Long C.X., Wang J. (2019). China's patent promotion policies and its quality implications. Sci. Publ. Pol..

[6] Feng Z., Wu Z. (2022). Local economy, asset location and REIT firm growth. J. R. Estate Finance Econ..

[7] Kekezi O., Klaesson J. (2020). Agglomeration and innovation of knowledge intensive business services. Ind. Innovat..

[8] Rodríguez-Rodríguez G., Ballesteros H.M., Martínez-Cabrera H., Vilela R., Pennino M.G., Bellido J.M. (2021). On the role of perception: understanding stakeholders' collaboration in natural resources management through the evolutionary theory of innovation. Sustainability.

[9] Juhász S., Lengyel B. (2018). Creation and persistence of ties in cluster knowledge networks. J. Econ. Geogr..

[10] Zhao S., Jiang Y., Peng X., Hong J. (2020). Knowledge sharing direction and innovation performance in organizations: do absorptive capacity and individual creativity matter?. Eur. J. Innovat. Manag..

[11] Howell A. (2020). Industry relatedness, FDI liberalization and the indigenous innovation process in China. Reg. Stud..

[12] Mate M.S., Harris R. (2018). The paradox of geographical proximity for innovators: a regional study of the Spanish agri-food sector. Land Use Pol..

[13] Huang K.F., Lin K.H., Wu L.Y., Yu P.H. (2015). Absorptive capacity and autonomous R&D climate roles in firm innovation. J. Bus. Res..

[14] Capello R., Caragliu A. (2018). Proximities and the intensity of scientific relations: synergies and nonlinearities. Int. Reg. Sci. Rev..

[15] Turkina E., Oreshkin B., Kali R. (2019). Regional innovation clusters and firm innovation performance: an interactionist approach. Reg. Stud..

[16] Torres de Oliveira R., Gentile-Lüdecke S., Figueira S. (2022). Barriers to innovation and innovation performance: the mediating role of external knowledge search in emerging economies. Small Bus. Econ..

[17] Arranz N., Arroyabe M., Li J., Fernandez de Arroyabe J.C. (2020). Innovation as a driver of eco‐innovation in the firm: an approach from the dynamic capabilities theory. Bus. Strat. Environ..

[18] Valdez L.E.J., Castillo M.V. (2021). Technological capabilities, open innovation, and eco-innovation: dynamic capabilities to increase corporate performance of SMEs. J. Open Innov.: Technol., Market, Complex..

[19] Marco-Lajara B., Sánchez-García E., Martínez-Falcó J., Poveda-Pareja E. (2022). Regional specialization, competitive pressure, and cooperation: the cocktail for innovation. Energies.

[20] Gordon P., Kourtit K. (2020). Agglomeration and clusters near and far for regional development: a critical assessment. Region. Sci. Policy Pract..

[21] Fang L. (2015). Do clusters encourage innovation? A meta-analysis. J. Plann. Lit..

[22] Ferras-Hernandez X., Nylund P.A. (2019). Clusters as innovation engines: the accelerating strengths of proximity. Eur. Manag. Rev..

[23] Stavroulakis P.J., Papadimitriou S., Tsioumas V., Koliousis I.G., Riza E., Kontolatou E.O. (2020). Strategic competitiveness in maritime clusters. Case Stud. Transp. Policy.

[24] Zhang Y. (2020). Being boutique in a hotel cluster: the benefits and threats of agglomeration. J. Strateg. Innov. Sustain..

[25] Holl A., Peters B., Rammer C. (2022). Local knowledge spillovers and innovation persistence of firms. Econ. Innovat. N. Technol..

[26] Losurdo F., Marra A., Cassetta E., Monarca U., Dileo I., Carlei V. (2019). Emerging specializations, competences and firms' proximity in digital industries: the case of London. Pap. Reg. Sci..

[27] Portugal M.F., Ribeiro F.S., Kramer B.C., Maccari E.A., Ritor H.C. (2012). Impact of the types of clusters on the innovation output and the appropriation of rents from innovation. J. Technol. Manag. Innovat..

[28] Kaličanin D., Gavrić O. (2014). The importance of clusters as drivers of competitive advantage of companies. Ekonomika preduzeća.

[29] Lu J., Tao Z. (2009). Trends and determinants of China's industrial agglomeration. J. Urban Econ..

[30] Williams C., Du J. (2014). The impact of trust and local learning on the innovative performance of MNE subsidiaries in China. Asia Pac. J. Manag..

[31] Zhang H. (2015). How does agglomeration promote the product innovation of Chinese firms?. China Econ. Rev..

[32] Boix-Domenech R., Capone F., Galletto V. (2022). Searching for “rare diamonds”? Industrial districts and innovation in Spain and Italy. Compet. Rev.: An International Business Journal.

[33] Uyarra E., Ramlogan R. (2012). The effects of cluster policy on innovation. https://www.nesta.org.uk/sites/default/files/the_effects_of_cluster_policy_on_innovation.pdf.

[34] Zheng W. (2010). A social capital perspective of innovation from individuals to nations: where is empirical literature directing us?. Int. J. Manag. Rev..

[35] Kratzer J., Meissner D., Roud V. (2017). Open innovation and company culture: internal openness makes the difference. Technol. Forecast. Soc. Change.

[36] Abu El-Ella N., Bessant J., Pinkwart A. (2016). Revisiting the honorable merchant: the reshaped role of trust in open innovation. Thunderbird Int. Bus. Rev..

[37] Pucci T., Brumana M., Minola T., Zanni L. (2020). Social capital and innovation in a life science cluster: the role of proximity and family involvement. J. Technol. Tran..

[38] Lins K.V., Servaes H., Tamayo A. (2017). Social capital, trust, and firm performance: the value of corporate social responsibility during the financial crisis. J. Finance.

[39] Tajpour M., Salamzadeh A., Salamzadeh Y., Braga V. (2021). Investigating social capital, trust and commitment in family business: case of media firms. J. Fam. Bus. Manag..

[40] König A., Kammerlander N., Enders A. (2013). The family innovator's dilemma: how family influence affects the adoption of discontinuous technologies by incumbent firms. Acad. Manag. Rev..

[41] Cao Y., Xiang Y. (2014). Study on the relationship among knowledge governance, knowledge sharing and employee innovation based on the mediating of social capital and the moderating of absorptive capacity in enterprises. Stud. Sci. Sci..

[42] Yoshida M., Gordon B.S., James J.D. (2021). Social capital and consumer happiness: toward an alternative explanation of consumer-brand identification. J. Brand Manag..

[43] Zhang H., Gupta S., Sun W., Zou Y. (2020). How social-media-enabled co-creation between customers and the firm drives business value? The perspective of organizational learning and social Capital. Inf. Manag..

[44] Kim B., Kim E., Foss N.J. (2016). Balancing absorptive capacity and inbound open innovation for sustained innovative performance: an attention-based view. Eur. Manag. J..

[45] Danquah M. (2018). Technology transfer, adoption of technology and the efficiency of nations: empirical evidence from sub Saharan Africa. Technol. Forecast. Soc. Change.

[46] Kokshagina O., Le Masson P., Bories F. (2017). Fast-connecting search practices: on the role of open innovation intermediary to accelerate the absorptive capacity. Technol. Forecast. Soc. Change.

[47] March J.G., Simon H.A. (1958).

[48] Cohen W.M., Levinthal D.A. (1990). Absorptive capacity: a new perspective on learning and innovation. Adm. Sci. Q..

[49] Lane P.J., Koka B.R., Pathak S. (2006). The reification of absorptive capacity: a critical review and rejuvenation of the construct. Acad. Manag. Rev..

[50] Zahra S.A., George G. (2002). Absorptive capacity: a review, reconceptualization, and extension. Acad. Manag. Rev..

[51] Scaringella L., Burtschell F. (2017). The challenges of radical innovation in Iran: knowledge transfer and absorptive capacity highlights—evidence from a joint venture in the construction sector. Technol. Forecast. Soc. Change.

[52] Albort-Morant G., Leal-Rodríguez A.L., De Marchi V. (2018). Absorptive capacity and relationship learning mechanisms as complementary drivers of green innovation performance. J. Knowl. Manag..

[53] Rezaei M.Z., Darwish T.K. (2016). Antecedents of absorptive capacity: a new model for developing learning processes. Learn. Organ..

[54] Kotlar J., De Massis A., Frattini F., Kammerlander N. (2020). Motivation gaps and implementation traps: the paradoxical and time‐varying effects of family ownership on firm absorptive capacity. J. Prod. Innovat. Manag..

[55] Miroshnychenko I., Strobl A., Matzler K., De Massis A. (2021). Absorptive capacity, strategic flexibility, and business model innovation: empirical evidence from Italian SMEs. J. Bus. Res..

[56] Aboelmaged M., Hashem G. (2019). Absorptive capacity and green innovation adoption in SMEs: the mediating effects of sustainable organisational capabilities. J. Clean. Prod..

[57] Sun P.Y., Anderson M.H. (2012). The combined influence of top and middle management leadership styles on absorptive capacity. Manag. Learn..

[58] Exposito-Langa M., Tomas-Miquel J.-V., Molina-Morales F.X. (2015). Innovation in clusters: exploration capacity, networking intensity and external resources. J. Organ. Change Manag..

[59] Li D., Wei Y.D., Miao C., Wu Y., Xiao W. (2019). Innovation, network capabilities, and sustainable development of regional economies in China. Sustainability.

[60] Parra-Requena G., Ruiz-Ortega M.J., Garcia-Villaverde P.M. (2013). Social capital and effective innovation in industrial districts: dual effect of absorptive capacity. Ind. Innovat..

[61] Solano G., Larrañeta B., Aguilar R. (2020). Absorptive capacity balance and new venture performance: cultivating knowledge from regional clusters. Technol. Anal. Strat. Manag..

[62] Resbeut M., Gugler P., Charoen D. (2019). Spatial agglomeration and specialization in emerging markets: economic efficiency of clusters in Thai industries. Compet. Rev.: An International Business Journal.

[63] Claver-Cortés E., Marco-Lajara B., Sánchez-García E., Seva-Larrosa P., Manresa-Marhuenda E., Ruiz-Fernández L., Poveda-Pareja E. (2020). A literature review on the effect of industrial clusters and the absorptive capacity on innovation. Int. J. Ind. Manuf. Eng..

[64] McCann P., Ortega-Argilés R. (2015). Smart specialization, regional growth and applications to European Union cohesion policy. Reg. Stud..

[65] Balle A.R., Oliveira M., Curado C.M.M. (2020). Knowledge sharing and absorptive capacity: interdependency and complementarity. J. Knowl. Manag..

[66] Wang X., Wang J., Zhang R. (2019). The optimal feasible knowledge transfer path in a knowledge creation driven team. Data Knowl. Eng..

[67] Hair J.F., Hult G.T.M., Ringle C., Sarstedt M.A. (2016).

[68] Armstrong J.S., Overton T.S. (1977). Estimating nonresponse bias in mail surveys. J. Market. Res..

[69] McCann B.T., Folta T.B. (2008). Location matters: where we have been and where we might go in agglomeration research. J. Manag..

[70] Kukalis S. (2010). Agglomeration economies and firm performance: the case of industry clusters. J. Manag..

[71] Diez-Vial I. (2011). Geographical cluster and performance: the case of Iberian ham. Food Pol..

[72] Boix R., Trullén J. (2010). Industrial districts, innovation and I-district effect: territory or industrial specialization?. Eur. Plann. Stud..

[73] Marco-Lajara B., Claver-Cortés E., Úbeda-García M., Zaragoza-Sáez P.D.C. (2016). Hotel performance and agglomeration of tourist districts. Reg. Stud..

[74] Prajogo D.I., Ahmed P.K. (2006). Relationships between innovation stimulus, innovation capacity, and innovation performance. R D Manag..

[75] Škerlavaj M., Song J.H., Lee Y. (2010). Organizational learning culture, innovative culture and innovations in South Korean firms. Expert Syst. Appl..

[76] Flatten T.C., Engelen A., Zahra S.A., Brettel M. (2011). A measure of absorptive capacity: scale development and validation. Eur. Manag. J..

[77] Finstad K. (2010). Response interpolation and scale sensitivity: evidence against 5-point scales. J. Usabil. Stud..

[78] Hair J.F., Sarstedt M., Pieper T.M., Ringle C.M. (2012). The use of partial least squares structural equation modeling in strategic management research: a review of past practices and recommendations for future applications. Long. Range Plan..

[79] Ringle C.M., Wende S., Becker J.M. (2015). *SmartPLS 3.* Bönningstedt, GE: SmartPLS. http://www.smartpls.com.

[80] Hair J.F., Hult G.T.M., Ringle C.M., Sarstedt M., Castillo Apraiz J., Cepeda Carrión G., Roldán J.L. (2019).

[81] Roldán J.L., Cepeda G. (2019).

[82] Martínez Ávila M., Fierro Moreno E. (2018). Aplicação da técnica PLS-SEM na gestão do conhecimento: uma abordagem técnica prática. RIDE. Revista Iberoamericana para la Investigación y el Desarrollo Educativo.

[83] Henseler J. (2018). Partial least squares path modeling: quo vadis?. Qual. Quantity.

[84] Henseler J., Hubona G., Ray P.A. (2016). Using PLS path modeling in new technology research: updated guidelines. Ind. Manag. Data Syst..

[85] Edwards J.R. (2001). Multidimensional constructs in organizational behavior research: an integrative analytical framework. Organ. Res. Methods.

[86] Henseler J. (2017). Bridging design and behavioral research with variance-based structural equation modeling. J. Advert..

[87] Sarstedt M., Hair J.F., Ringle C.M., Thiele K.O., Gudergan S.P. (2016). Estimation issues with PLS and CBSEM: where the bias lies. J. Bus. Res..

[88] Ringle C.M., Sarstedt M., Straub D.W. (2012). Editor's comments: a critical look at the use of PLS-SEM. MIS Q..

[89] Hu L.T., Bentler P.M. (1998). Fit indices in covariance structure modeling: sensitivity to underparameterized model misspecification. Psychol. Methods.

[90] Hair J.F., Risher J.J., Sarstedt M., Ringle C.M. (2019). When to use and how to report the results of PLS-SEM. Eur. Bus. Rev..

[91] Chin W.W. (1998). The partial least squares approach to structural equation modeling. Mod. Methods Bus. Res..

[92] Sarstedt M., Wilczynski P., Melewar T.C. (2013). Measuring reputation in global markets—a comparison of reputation measures' convergent and criterion validities. J. World Bus..

[93] Hair J.F., Babin B.J., Anderson R.E., Black W.C. (2019).

[94] Dijkstra T.K., Henseler J. (2015). Consistent partial least squares path modeling. MIS Q..

[95] Henseler J., Ringle C.M., Sarstedt M. (2015). A new criterion for assessing discriminant validity in variance-based structural equation modeling. J. Acad. Market. Sci..

[96] Kline R.B., Malcolm W., Vogt W.P. (2011). The SAGE Handbook of Innovation in Social Research Methods.

[97] Cohen J. (1988).

[98] Presutti M., Boari C., Majocchi A., Molina-Morales X. (2019). Distance to customers, absorptive capacity, and innovation in high-tech firms: the dark face of geographical proximity. J. Small Bus. Manag..

[99] Molina-Morales F.X., Martínez-Fernández M.T. (2009). Too much love in the neighborhood can hurt: how an excess of intensity and trust in relationships may produce negative effects on firms. Strat. Manag. J..

